# Stroke recovery and lesion reduction following acute isolated bilateral ischaemic pontine infarction: a case report

**DOI:** 10.1186/1756-0500-7-728

**Published:** 2014-10-16

**Authors:** Ourania Varsou, Michael S Stringer, Catarina Dinis Fernandes, Christian Schwarzbauer, Mary Joan MacLeod

**Affiliations:** Aberdeen Biomedical Imaging Centre, University of Aberdeen, Foresterhill, Aberdeen, AB25 2ZD UK; Department of Medicine and Therapeutics, University of Aberdeen, Foresterhill, Aberdeen, AB25 2ZD UK

**Keywords:** Acute stroke, Pontine stroke, Pons, Magnetic resonance imaging, MRI

## Abstract

**Background:**

Although pontine strokes account for a small percentage of all ischaemic events, they can be associated with significant initial disability. These lesions may be missed on computed tomography and therefore magnetic resonance imaging is generally preferred for the assessment of brainstem strokes. The aetiopathogenesis of isolated pontine infarcts, not due to a significant compromise (occlusion or dissection) in the vertebrobasilar territory, still remains to be fully characterised. These strokes present with different symptoms, depending on the lesion location and size, partly reflecting the anatomical variability of the vertebrobasilar vessels. Progressive neurological deterioration is relatively common and has been associated with the extension of such lesions. However, many patients with significant infarcts in the pons will do well in the future and initial diffusion-weighted imaging may not add useful prognostication to the clinical assessment. We discuss here a case where an initially progressive presentation was associated with a marked improvement in both clinical and radiological assessments at 42 days.

**Case presentation:**

A 49-year-old white British man presented with left-sided weakness, incoordination, unsteadiness, cerebellar ataxic dysarthria and dysphonia. A baseline magnetic resonance imaging scan with diffusion-weighted imaging, T_1_-weighted and T_2_-weighted sequences showed an acute bilateral pontine infarct. On a repeat scan at 42 days, there was a 57.5% decrease in the size of the lesion on the high-resolution three-dimensional T_1_-weighted image and a corresponding improvement in the symptoms and the clinical assessments of this patient. The reduction in infarct size was also comparable to the decrease calculated between the baseline diffusion-weighted and the follow-up fluid attenuated inversion recovery sequences.

**Conclusion:**

This case report discusses the significant clinical improvement and corresponding lesion reduction in a patient that presented with worsening neurological symptoms and was diagnosed with acute bilateral ischaemic pontine infarction. Further studies, utilising structural and functional magnetic resonance imaging with follow-up scans, are needed to provide better insights into the underlying aetiopathology and recovery mechanisms of pontine stroke. These will help define the relationship between imaging parameters and outcome allowing for better prognosis along with the development of relevant rehabilitation programs for this group of patients.

**Electronic supplementary material:**

The online version of this article (doi:10.1186/1756-0500-7-728) contains supplementary material, which is available to authorized users.

## Background

Pontine strokes account for approximately 7% of all ischaemic events [1] and may present with progressive symptoms that cause clinical concern. Patients tend to do generally well, if a bilateral lesion is not present [2]. In addition, pontine infarcts may be missed when computed tomography (CT) is the imaging modality used and clinicians should have a high index of suspicion in patients with pure motor symptoms [3]. The aetiopathogenesis of pontine infarction in the absence of vertebrobasilar occlusion or dissection is still not fully characterised. [4]. Both small vessel (lipohyalinosis) and large vessel disease (atherosclerosis), especially of the basilar artery branches in the latter case, have been proposed as possible causes of pontine ischaemic strokes [2, 4–6]. An imaging study also suggested basilar artery stenosis as a potential aetiology for this condition [7]. Patients with isolated pontine infarcts can present with a wide range of different symptoms, depending on the portion of the pons that has been affected and the size of the lesion, partly reflecting the highly variable vertebrobasilar vessel anatomy [8]. Furthermore, neurological deterioration is relatively common even in the absence of a basilar thrombus, and has been associated with the enlargement of the pontine lesions. This is most likely a dynamic process with interacting haemodynamic, metabolic, inflammatory and cellular factors [4].

## Case presentation

A 49-year-old, right-handed white British, conscious man arrived at the emergency department (ED) in the summer of 2013. He gave a history of waking up the previous day with mild left-sided weakness, affecting both his upper and lower limbs, along with a minor degree of unsteadiness on his feet. At that point, he did not experience any facial weakness, sensory symptoms, seizures or a headache. The left-sided weakness had somewhat improved on the date the patient presented to ED, but he had developed slurred speech that was present since waking up that morning. His past medical history included hypertension, hypercholesterolaemia and type II diabetes mellitus since the age of 42 that was treated with oral hypoglycaemic agents. His regular medications included aspirin, metformin, sitagliptin, dapagliflozin, rosuvastatin, bezafibrate and irbesartan. Social history was unremarkable, with no current or past use of tobacco or alcohol. On admission, his initial recordings included a respiratory rate of 15 breaths per minute, a pulse of 69 beats per minute, a blood pressure of 165/91 mmHg, a temperature of 35.4°C, a capillary blood glucose measurement of 13.6 mmol/L and a Glasgow Coma Scale (GCS) of 15 out of 15 (spontaneous eye opening with 4 out of 4, normal motor responses with 6 out of 6 and normal verbal responses with 5 out of 5). Initial examination at the ED revealed a mild left-sided weakness in both upper and lower limbs with 4 out of 5 in the Medical Research Council (MRC) muscle power grading scale, slight left facial weakness with loss of the nasolabial fold and dysarthria. An urgent non-contrast brain CT was performed that showed a small area of decreased attenuation in the anterior limb of the right internal capsule. This was in keeping with an established lacunar infarct. The symptoms experienced by the patient, the examination findings and the initial imaging results were suggestive of a lacunar ischaemic stroke. Following discussion with the on-call stroke team, the patient was started on aspirin 300 mg once daily and then admitted to the Acute Stroke Unit for further inpatient management.

**Figure 1:**
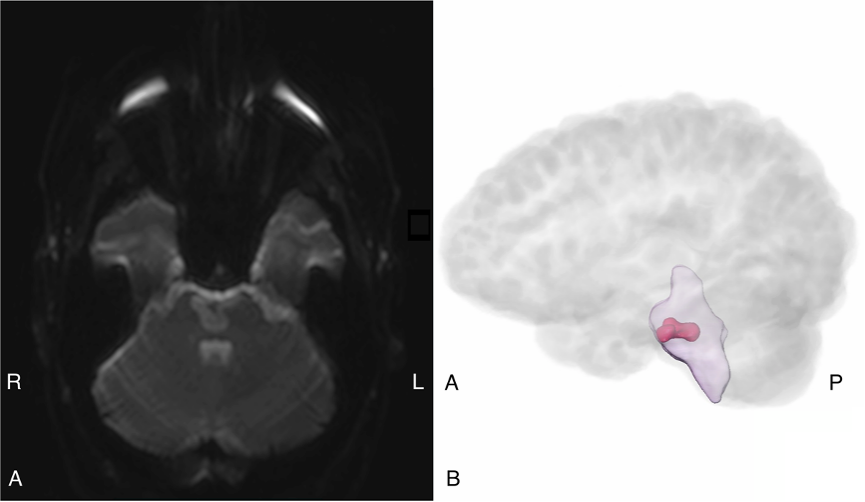
**Diffusion-weighted imaging of the pontine infarct at baseline. (A)** Axial diffusion-weighted imaging of the pontine infarct. **(B)** The pontine infarct shown on a sagittal three-dimensional representation of the brainstem with the brain incorporated in the image (brain outlined in light grey, brainstem shown in purple and pontine infarct marked in red).

When reassessed the following morning, the patient was still experiencing left-sided weakness affecting his upper and lower limbs, mild left facial asymmetry on smiling along with loss of the corresponding nasolabial fold and dysarthria that was classified as cerebellar ataxic in nature. He had also developed some elements of dysphonia. In addition, he was noted to have poor left-sided coordination with mild ataxia during the finger-to-nose and heel-to-shin test. Due to the on-going symptoms and the development of new signs, some of which suggested brainstem pathology, the patient had a CT angiogram from the aortic arch up to and including the circle of Willis. This showed that both carotid and vertebral arteries were patent, with the right vertebral artery being dominant. There was a minor narrowing of the intracranial portion of the right vertebral artery without an occlusion or dissection being present, and no evidence of basilar artery thrombosis. Following this investigation, a brain magnetic resonance imaging (MRI) scan was performed the following day (four days after the original onset of symptoms). The scan showed high signal in the anterior pons on both sides of the midline that was extending posteriorly in the diffusion-weighted imaging (DWI) axial trace image. This was accompanied by a low signal in the calculated apparent diffusion coefficient (ADC) map, indicating diffusion restriction, in keeping with an acute infarction. Ischaemic changes were also noted in the right caudate head and putamen without any corresponding DWI changes suggestive of an acute infarct. Finally, there were multiple small hyperintensities in the basal ganglia, the thalamus and the subcortical along with the deep white matter that were representative of small vessel disease. The overall appearances from the MRI were suggestive of an acute isolated pontine infarction (Figure 1). Following this scan, the patient was commenced on dual antiplatelet treatment with aspirin and clopidogrel. In addition to the on-going medical and nursing care, the patient received daily input from the physiotherapist, speech and language therapist, occupational therapist, dietician and pharmacist for the duration of his inpatient stay.

**Figure 2:**
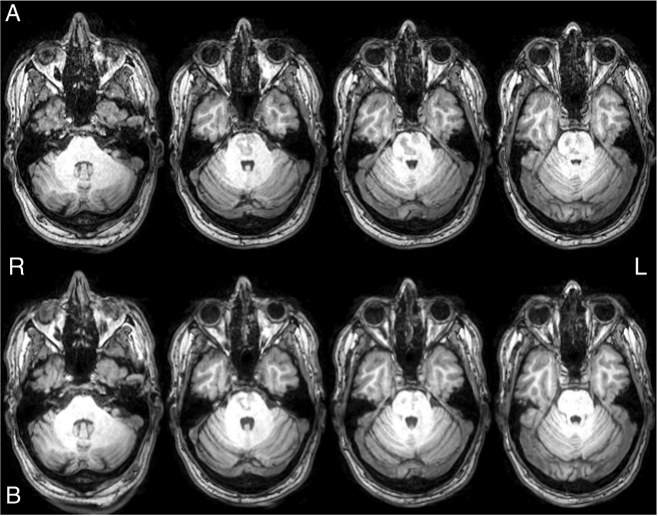
**Baseline and follow-up T**_**1**_ **-weighted structural images. (A)** Axial T_1_-weighted images of the pontine infarct from the baseline magnetic resonance imaging scan. **(B)** Axial T_1_-weighted images of the pontine infarct from the follow-up magnetic resonance imaging scan.

**Figure 3:**
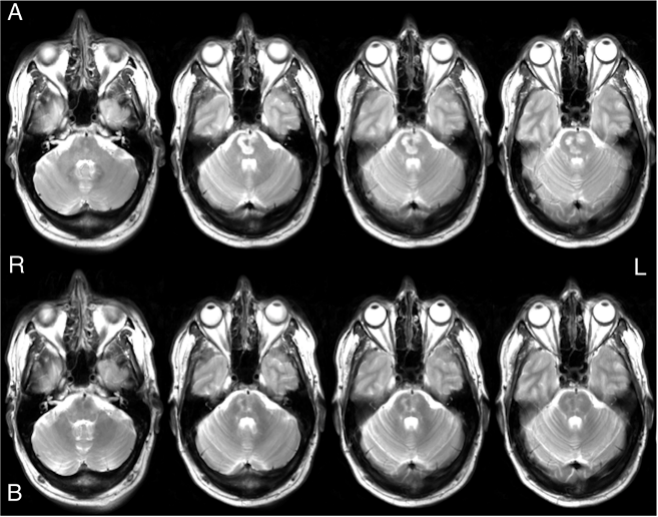
**Baseline and follow-up T**_**2**_ **-weighted structural images. (A)** Axial T_2_-weighted images of the pontine infarct from the baseline magnetic resonance imaging scan. **(B)** Axial T_2_-weighted images of the pontine infarct from the follow-up magnetic resonance imaging scan.

**Figure 4:**
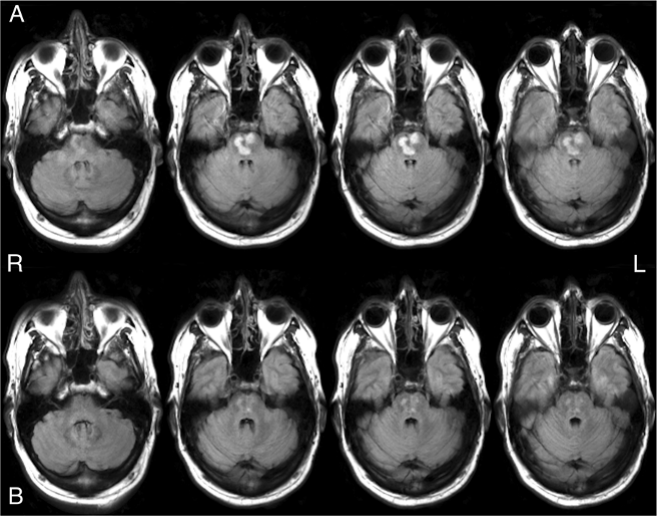
**Baseline and follow-up fluid attenuated inversion recovery structural images. (A)** Axial fluid attenuated inversion recovery images of the pontine infarct from the baseline magnetic resonance imaging scan. **(B)** Axial fluid attenuated inversion recovery images of the pontine infarct from the follow-up magnetic resonance imaging scan.

At nine days from admission to ED, the patient was transferred to the stroke rehabilitation unit of a local community hospital for continuing stroke specific care. As an inpatient at the community hospital, he received tailored one-to-one physiotherapy input with a total of 13 therapy hours over a period of 22 days. Following discharge, he continued to attend weekly physiotherapy sessions on an outpatient basis and the overall time spent in therapy for six months was close to 20 hours. Approximately 45 days after the patient initially presented to ED, he attended for review and a follow-up brain MRI. At that point, his left-sided weakness and the speech problems had significantly improved. His blood pressure was not elevated (122/84 mmHg) and was generally feeling better. The follow-up MRI revealed no new lesions and the pontine infarct had visibly reduced in size on the T_1_-weighted, T_2_-weighted and fluid attenuated inversion recovery (FLAIR) sequences (Figures 2, 3 and 4) that was radiologically in keeping with a resolving infarct. Currently, the patient has improved sufficiently to return to work.

## Discussion

Over a period of approximately 42 days from the baseline to the follow-up MRI, the percentage reduction in the volume of the pontine infarct on T_1_-weighted imaging was 57.9% according to a physician (OV) and 57.0% from a biomedical engineer (CDF), with an average of 57.5% volume decrease between these two raters. The T_1_-weighted image was specifically chosen for the analysis, as it was the sequence with the highest resolution that was also acquired in all three anatomical planes (see Additional file 1). Furthermore, the analysis software used to create the mask of the brainstem works best with this sequence, as it creates the masks based on results from a large database of manually segmented T_1_-weighted images. The reduction in infarct size was also comparable to the decrease calculated between the baseline DWI and the follow-up FLAIR and mirrored the improvement noted in clinical assessments along with the symptoms reported by the patient. It is noteworthy that over five weeks, under standard stroke rehabilitation care, there was more than 50% decrease in the size of the pontine infarct. This lesion reduction and the clinical improvement are likely to reflect the dynamic process of the following parameters that contribute to pontine stroke: haemodynamic, metabolic, inflammatory and cellular factors. It is also of note that the patient has managed to resume his usual activities and returned to work within approximately six months from the original onset of symptoms.

Although pontine infarction accounts for a small percentage of all ischaemic events [1], it is an important and interesting condition, especially as its aetiopathogenesis currently remains unclear [4]. The vascular centrencephalon, however, may provide a further insight into the underlying pathophysiology of infarcts that occur in the pons. This concept refers to the areas of the brain, including the brainstem, that are supplied by end arteries arising perpendicularly from major vessels of the anterior and posterior circulation without any collaterals. Due to this rigid anatomical arrangement, the end arteries are particularly vulnerable to injury (narrowing and rupture of the vessel wall) from various mechanical stressors such as hypertension [9].

Most patients with pontine infarcts tend to have generally good motor outcomes [10], if bilateral pontine lesions are not present [2], but the overall functional prognosis does depend on a number of factors such as age and cognitive function [11]. Depending on the location and extent of the lesion, patients can experience significant disability that places a heavy burden on sufferers, their relatives, the clinicians and healthcare resources. Progression of motor weakness in this condition has also been associated with the topography of the infarct, with lesions in the lower pons being an independent prognostic factor [12]. Interestingly, several motor recovery mechanisms have been proposed in pontine infarction, including peri-lesional reorganisation via the aberrant pyramidal tract through the medial lemniscus in the brainstem, ipsilateral motor pathways and recovery via the spared corticospinal tract (CST) that is passing from the pons [9]. Finally, our findings agree with the results of a systematic review questioning the accuracy of initial MRI in reflecting final infarct size. Specifically, there is increasing evidence that the reliability of DWI to reflect infarct core is less robust than originally thought, and that a considerable number of infarcts are reduced in size on repeat imaging [13]. The lesion reduction along with the improvement noted following the initial clinical worsening may be attributable to the development of cerebral oedema associated with the acute infarct during the early stages which then decreases over time.

## Conclusion

This case report discusses the significant clinical improvement and lesion reduction noted over a period of approximately five weeks in a patient that presented with worsening neurological symptoms and was found to have an acute isolated bilateral ischaemic pontine infarct. Further studies of pontine stroke, correlating clinical features with MRI over time, will help clinicians give informed advice regarding prognosis. These studies should be ideally characterised by unique multi-planar capabilities, excellent spatial resolution and advanced multimodal scanning protocols that can incorporate both structural and functional sequences.

## Consent

Written informed consent was obtained from the patient for publication of this Case Report and any accompanying images. A copy of the written consent is available for review by the Editor-in-Chief of this journal.

## Electronic supplementary material

Additional file 1: **Additional methods.** (DOCX 26 KB)

## Abbreviations

ADC: Apparent diffusion coefficient
CST: Corticospinal tract
CT: Computed tomography
DWI: Diffusion-weighted imaging
ED: Emergency department
FLAIR: Fluid attenuated inversion recovery
GCS: Glasgow coma scale
MRC: Medical research council
MRI: Magnetic resonance imaging.
