# Supplementary material for: Stroke recovery and lesion reduction following acute isolated bilateral ischaemic pontine infarction: a case report
Source: BMC Res Notes. 2014 Oct 16;7:728. doi: 10.1186/1756-0500-7-728 (PMC4203895; doi:10.1186/1756-0500-7-728)
Supplement: Supplementary file 1 — Additional file 1: Additional methods. (DOCX 26 KB) [file 13104_2014_3243_MOESM1_ESM.docx]

#### Additional file

**Stroke recovery and lesion reduction following acute isolated bilateral ischaemic pontine infarction: a case report**

**Ourania Varsou, Michael S Stringer, Catarina Dinis Fernandes, Christian Schwarzbauer and Mary Joan Macleod**

**Additional clinical assessments**

The pre-morbid Oxford Handicap Scale was 0 (no symptoms) out of 5 and the patient was leading an active professional and personal life. On admission to the Acute Stroke Unit, he was able to lift both arms off the bed and he was also able to walk without help. The Hodkinson Abbreviated Mental Test (AMT) Score was also 10 out of 10. When the patient attended for his baseline magnetic resonance imaging (MRI), the modified Rankin Scale (mRS) was 2 (slight disability with inability to carry out all usual daily activities, but able to attend to his own needs) out of 6, the National Institutes of Health Stroke Scale (NIHSS) was 3 out of 42 and the Montreal Cognitive Assessment (MoCA English language original version 7.1) was 24 out of 30. Although the mRS was still 2 at the follow-up MRI, the NIHSS had decreased to 1 and the MoCA score was 30. The above clinical assessments suggest that the patient was improving when compared to his first MRI visit.

The Scheltens semiquantitative visual rating scale was also used to grade white matter hyperintensities on the T_2_-weighted and the fluid attenuated inversion recovery (FLAIR) structural sequences. The Scheltens scale provides information based on the number and size of the hyperintensities at different anatomical locations [1]. The T_2_-weighted and FLAIR sequences, from both scanning sessions, were independently assessed for hyperintensities by a trained and experienced rater (OV). Specifically, the white matter along with periventricular hyperintensities were primarily scored on the FLAIR sequence and the grey matter along with the infratentorial hyperintensities were mainly graded on the T_2_-weighted sequence. The total Scheltens score for the baseline MRI was 33 (10 for white matter, 10 for grey matter, 8 for infratentorial and 5 for periventricular hyperintensities) and that had slightly increased to 35 (10 for white matter, 12 for grey matter, 8 for infratentorial and 5 for periventricular hyperintensities) at the follow-up MRI. This small increase was probably due to the previous acute ischaemic changes in the grey matter changing to hyperintensities over time, which is not an uncommon radiological finding.

**Imaging data acquisition**

The imaging data were acquired on a Philips Achieva 3.0 Tesla X-series MRI scanner (Philips Healthcare, Best, The Netherlands; <http://www.philips.com/global/index.page>), using a Siemens 32-channel receive-only phased-array head coil (Siemens Medical Systems, Iselin, NJ; <http://www.healthcare.siemens.co.uk>), at the Aberdeen Biomedical Imaging Centre. The MRI neurovascular scanning protocol, for both the baseline and follow-up visits, consisted of the following sequences: i) a high-resolution three-dimensional T_1_-weighted turbo field gradient echo structural sequence (total acquisition time of 5 min and 35 s, repetition time of 8200 ms, echo time of 3.8 ms, flip angle of 8˚, 240 × 240 × 160 mm^3^ field of view, 1.0 × 1.0 × 1.0 mm^3^ voxel size, 160 slices); ii) a T_2_*-weighted steady state free precession gradient echo structural sequence (total acquisition time of 2 min and 9 s, repetition time of 706 ms, echo time of 16.1 ms, flip angle of 18˚, 230 × 182 × 131 mm^3^ field of view, 1.0 × 1.0 mm^2^ voxel size, 24 slices); iii) a T_2_-weighted short-τ inversion recovery spin echo structural sequence (total acquisition time of 3 min and 6 s, repetition time of 3000 ms, echo time of 80 ms, inversion time τ of 100-150 ms, flip angle of 90˚, 230 × 184 × 129 mm^3^ field of view, 0.8 × 0.8 mm^2^ voxel size, 26 slices); iv) a FLAIR spin echo structural sequence (total acquisition time of 5 min and 52 s, repetition time of 11000 ms, echo time of 125 ms, refocusing angle of 120˚, 230 × 230 × 144 mm^3^ field of view, 0.7 × 0.9 mm^2^ voxel size, 29 slices); and v) a diffusion-weighted imaging (DWI) functional sequence (total acquisition time of 1 min and 31 s, b value of 1000, repetition time of 2859 ms, echo time of 76 ms, flip angle of 90˚, 230 × 230 × 131 mm^3^ field of view, 1.5 × 1.5 mm^2^ voxel size, 24 slices, SENSE parallel imaging method). An experienced neuroradiologist interpreted all sequences from both the baseline and follow-up MRI.

**Imaging data preprocessing**

Preprocessing of the imaging data was carried out through FSL (FMRIB Software Library Version 5.0; <http://fsl.fmrib.ox.ac.uk/fsl/fslwiki/>). The images were kept in the native subject space throughout the analysis, as comparison across different participants was not required in this case report and also to prevent the risk of any potential spatial normalization artefacts arising from registering to the Montreal Neurological Institute (MNI) stereotactic template. The T_1_-weighted image from the follow-up MRI was initially co-registered to the T_1_-weighted image from the baseline MRI using FLIRT (FMRIB's Linear Image Registration Tool; <http://fsl.fmrib.ox.ac.uk/fsl/fslwiki/FLIRT>) [2-4], after which both were reoriented to match the FSL standard template for visualization purposes. FLIRT provides an automated and robust method of applying linear registration to images, in this case between two different scanning sessions, allowing direct comparison between the scans in the later analysis. FIRST (FMRIB Integrated Registration and Segmentation Tool; <http://fsl.fmrib.ox.ac.uk/fsl/fslwiki/FIRST>) was then used in order to obtain a mask for the brainstem, using the T_1_-weighted image from the baseline MRI. Based on a model constructed from a database of manually labelled images, the most probable shape of subcortical structures can be obtained from the observed intensities in the T_1_-weighted image [5]. The T_1_-weighted images from both scanning sessions were then brain extracted to remove the skull, neck and other extraneous tissue that was out-with the brain using BET (Brain Extraction Tool Version 2.1; <http://fsl.fmrib.ox.ac.uk/fsl/fsl-4.1.9/bet2/index.html>) [6]. Removing non-brain tissue improves the accuracy of segmentation and reduces the risk of errors in later segmentation steps [6].

**Imaging data analysis**

FAST (FMRIB's Automated Segmentation Tool Version 4.0; <http://fsl.fmrib.ox.ac.uk/fsl/fslwiki/FAST>) was used to carry out segmentation on both T_1_-weighted images, from the baseline and follow-up MRI, in order to separate the brain into different tissue-type classes (cerebrospinal fluid, grey matter and white matter). FAST segments images using an automated process that provides a robust way of tissue segmentation [7]. It was found through experimentation that the program did not separate the lesion into a distinct tissue class, with up to five different tissue classes generated, but it was noted that the lesion was included in the grey matter images from each scanning session that were then used for further analysis. The grey matter image from the baseline MRI was loaded into FSLView (FSLView Version 3.1; <http://fsl.fmrib.ox.ac.uk/fsl/fslview/>) along with the brainstem mask, within which two suitably qualified blinded raters (OV and CDF) independently created a mask of the infarct by manual segmentation. Lastly, the routine “fslstats” in FSL was used to determine the volume of the infarct within the two grey matter images by applying the mask created previously. A percentage reduction in the volume of the pontine infarct between the baseline and follow-up MRI was then calculated. Finally, the same approach was adopted to calculate the lesion reduction by a blinded rate (MSS) between the baseline diffusion-weighted image (DWI) and the follow-up fluid attenuated inversion recovery (FLAIR) sequence.

**References**

1. Scheltens P, Barkhof F, Leys D, Pruvo JP, Nauta JJ, Vermersch P, Steinling M, Valk J: **A semiquantative rating scale for the assessment of signal hyperintensities on magnetic resonance imaging.** *J Neurol Sci* 1993, **114:**7-12.
2. Greve DN, Fischl B: **Accurate and robust brain image alignment using boundary-based registration.** *NeuroImage* 2009, **48**:63-72.
3. Jenkinson M, Smith S: **A global optimisation method for robust affine registration of brain images.** *Med Image Anal* 2001, **5**:143-156.
4. Jenkinson M, Bannister P, Brady M, Smith S: **Improved optimization for the robust and accurate linear registration and motion correction of brain images.** *NeuroImage* 2002, **17**:825-841.
5. Patenaude B, Smith SM, Kennedy DN, Jenkinson M: **A Bayesian model of shape and appearance for subcortical brain segmentation.** *NeuroImage* 2011, **56**:907-922.
6. Smith SM: **Fast robust automated brain extraction.** *Hum Brain Map* 2002, **17**:143-155.
7. Zhang Y, Brady M, Smith S: **Segmentation of brain MR images through a hidden Markov random field model and the expectation-maximization algorithm.** *IEEE* *Trans Med Imaging* 2001, **20**:45-57.
